# Synthesis of Silver, Gold, and Platinum Doped Zinc Oxide Nanoparticles by Pulsed Laser Ablation in Water

**DOI:** 10.3390/nano12193484

**Published:** 2022-10-05

**Authors:** Rafaela Radičić, Dejan Maletić, Damjan Blažeka, Julio Car, Nikša Krstulović

**Affiliations:** Institute of Physics, Bijenička cesta 46, 10000 Zagreb, Croatia

**Keywords:** zinc oxide (ZnO), doped ZnO, silver (Ag) doped ZnO, gold (Au) doped ZnO, platinum (Pt) doped ZnO, pulsed laser deposition (PLD), pulsed laser ablation in liquid (PLAL), nanoparticles

## Abstract

In this paper, we propose a simple two-step method for the synthesis of Ag, Au, and Pt-doped ZnO nanoparticles. The method is based on the fabrication of targets using the pulsed laser deposition (PLD) technique where thin layers of metals (Ag, Pt, Au) have been deposited on a metal-oxide bulk substrate (ZnO). Such formed structures were used as a target for the production of doped nanoparticles (ZnO: Ag, ZnO: Au, and ZnO: Pt) by laser ablation in water. The influence of Ag, Au, and Pt doping on the optical properties, structure and composition, sizing, and morphology was studied using UV-Visible (UV-Vis) and photoluminescence (PL) spectroscopies, X-ray diffraction (XRD), X-ray photoelectron spectroscopy (XPS), scanning electron microscopy (SEM), and transmission electron microscopy (TEM), respectively. The band-gap energy decreased to 3.06, 3.08, and 3.15 for silver, gold, and platinum-doped ZnO compared to the pure ZnO (3.2 eV). PL spectra showed a decrease in the recombination rate of the electrons and holes in the case of doped ZnO. SEM, TEM, and AFM images showed spherical-shaped nanoparticles with a relatively smooth surface. The XRD patterns confirm that Ag, Au, and Pt were well incorporated inside the ZnO lattice and maintained a hexagonal wurtzite structure. This work could provide a new way for synthesizing various doped materials.

## 1. Introduction

In nanotechnology, nanoparticles—particles in the range of 1 to 100 nm in diameter—have a significant role due to their exceptional magnetic, electrical, mechanical, optical, and electronic properties with respect to the bulk materials [1]. These unique properties allow the use of nanoparticles in energy harvesting [2], sensing [3], optics [4], photocatalysis [5], cosmetics [6], medicine [7], and biology [8]. Metal oxide nanoparticles excel as the most used nanomaterials due to their various properties, such as being adsorbents to heavy metals or having unique opto-electrical properties, catalytic sensitivity, and selective activity [9].

Beside pure metallic nanoparticles, two-component (alloyed) and metal-oxide nanoparticles represent advanced functional materials, which have high impact in a wide variety of applications in science and technology [10]. Such nanoparticles can be fabricated directly from alloyed targets by PLAL in a form of core-shell nanoparticles [11], alloyed nanoparticles [12], or as hybrid nanostructures where PLAL nanoparticles are adsorbed on the support or nanostructured surfaces [13,14].

Among metal oxide nanoparticles, zinc oxide (ZnO) is one of the most prominent and widely used materials for gas and chemical sensors [15], optical and electrical devices [16], solar cells [17], water treatment [18], antimicrobial activity [19], food packaging [20], and drug delivery [21]. ZnO is an inorganic n-type semiconductor with a direct bandgap of 3.37 eV and binding energy of 60 meV at room temperature. It has unique physicochemical properties, such as piezoelectricity [22], pyroelectric effects [23], good electron transport [24], and photo- and sono-catalytic activities [25]. ZnO strongly absorbs UVA (315–400 nm) and UVB (280–315 nm) light [19], which is why it is one of the most effective sun protectors available. At ambient conditions, ZnO has a hexagonal wurtzite structure with intrinsic defects—O vacancies and Zn interstitials—resulting in n-type conductivity. Introducing a new element in the crystal structure of ZnO leads to the enhancement of the electrical and optical properties and broadens the area of its application [26]. For n-type doping, we can use group-III (Al, Ga, In), as substitutional elements for Zn, and group-VII (Cl, I), as substitutional elements for O. The big challenge is to obtain p-type doping in wide-gap semiconductors such as ZnO because of the native defects, hydrogen impurities, low solubility of the dopant, and deep impurity level [27]. Known acceptors for p-type doping are Group-I elements (Li, Na, K), Group-V elements (N, P, As), silver, copper, and Zn vacancies.

Various methods are developed for ZnO doping, such as chemical vapor deposition (CVP) [28], sol-gel [29], atomic layer deposition [30], pulsed laser deposition (PLD) [31], a wet chemical method [32], etc. Drawbacks of chemical methods are the usage of various chemicals in the synthesis process, toxic by-products, stabilizers, and capping agents, while high-vacuum methods can be complicated to handle and expensive. Pulsed laser ablation in liquid (PLAL) gained a lot of attention due to its fast production of nanoparticles with production rates of several grams per hour [33], simplicity, and effectiveness. In the PLAL technique, a pulsed laser beam ablates a metal plate immersed in a liquid where plasma is formed on the surface of the metal plate in the focus of the high-power laser beam. There is an energy exchange from the plasma to the liquid, where a cavitation bubble forms. Eventually, particles are released from the cavitation bubble into the surrounding liquid. Produced nanoparticles have high purity (ligand-free), and their size and shape depend on the ablation parameters (laser wavelength and fluence, repetition rate, ablation time, and liquid environment) [34]. Furthermore, there is no limit on the type of produced nanoparticles because every metal target can be ablated. Since there are no toxic by-products, the method can be classified as eco-friendly.

According to the literature, researchers successfully produced doped ZnO using a nanosecond or femtosecond pulsed laser in a liquid environment. Sahoo et al. [35] generated Mg-doped ZnO nanoparticles in ethanol using a Ti: Sapphire femtosecond laser. The Mg: ZnO target was prepared by grinding together the MgO and ZnO powders. After that, the mixture was compressed and sintered at 1200 °C for 24 h. Also, Chelnokov et al. [36] produced Mg-doped ZnO nanoparticles where the target was prepared from mixtures of Zn and Mg acetylacetonates. The mixture was manually mixed and heated for one hour at 130 °C to evaporate water and then calcined for three hours at 350 °C to destroy organics. Lastly, the mixture was compressed and sintered for two days at 700 °C. The Mg: ZnO nanoparticles were synthesized in ethanol by Ti: Sapphire femtosecond laser. Qin et al. [37] generated Cu-doped ZnO quantum dots using an ns-Nd: YAG laser in Polyvinylpyrrolidone (PVP) aqueous solution. The Zn/Cu composite targets were synthesized by a chemical replacement method. Yudasari et al. [38] employed an ns-Nd: YAG laser for Fe-doped ZnO nanoparticle production using the Zn and Fe targets. Firstly, the Zn plate was ablated in pure water. Then, the Zn plate was replaced with a Fe plate. Lastly, Fe: ZnO nanoparticles were generated by ablating the Fe plate in the ZnO colloidal solution. With this method, Anugrahwidya et al. [39] produced Ag-doped ZnO nanoparticles, and Khashan et al. [40] synthesized indium-doped ZnO. Krstulović et al. [41] used ns-Nd: YAG laser for ZnO: Al_2_O_3_ target ablation in MiliQ water and, consequently, produced Al-doped ZnO nanoparticles. To our knowledge, PLAL was not used for Au and Pt-doped ZnO nanoparticle synthesis.

In this work, we present a newly developed synthesis method for Ag, Au, and Pt-doped ZnO that combines the PLD and the PLAL processes. With PLD, we created ZnO-X (X-Au, Ag, Pt) targets for the ablation process in water using ns-Nd: YAG laser. The advantage of this target synthesis is simplicity and fast production compared to the previous methods.

## 2. Experimental Procedure

### 2.1. Material Preparation

**Figure 1:**
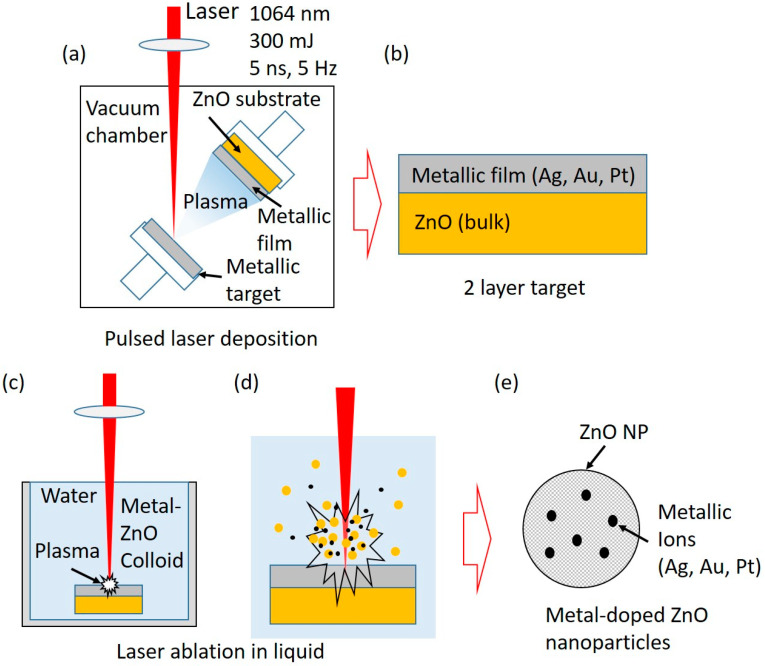
Two-step synthesis method of Ag, Au, and Pt-doped ZnO nanoparticles. First step is (**a**) pulsed laser deposition (PLD) of Ag, Au, and Pt on the ZnO substrate. As result, (**b**) a two-layered target (ZnO-metallic film) is formed. In the second step, (**c**,**d**) a two-layer target is ablated in water, forming (**e**) Ag, Au, and Pt-doped ZnO NPs.

The X: ZnO (X-Ag, Au, Pt) doped nanoparticles were synthesized in a two-step process shown in Figure 1. Firstly, we deposited a thin layer of Ag, Au, and Pt (purity > 99.9%, GoodFellow, Huntingdon, UK) on ZnO ceramic (purity > 99.99%, GoodFellow, Huntingdon, UK) substrates using the pulsed laser deposition (PLD) method in order to obtain targets for the PLAL process (Figure 1a). The deposition was performed in a vacuum (10^−4^ mbar) while the target and substrate were rotated to avoid the drilling of the target and to ensure homogeneous film deposition on the substrate. The laser pulse number was set to 2000 for all samples. In the second step, ZnO-X (X-Ag, Au, Pt) targets were used in the PLAL process for obtaining X: ZnO (X-Ag, Au, Pt) doped nanoparticle solutions (Figure 1c,d). The targets were immersed 2.5 cm under water in a glass beaker filled with 25 mL MiliQ water. The ablation process was carried out by an Nd: YAG (Quantel, Brilliant, Les Ulis, France) laser with the wavelength and pulse output energy of 1064 nm and 300 mJ, respectively. The pulse duration and repetition rate were 5 ns and 5 Hz, while the ablation time was 6 min and 40 s (2000 pulses). The energy delivered to the sample was approximately 120 mJ per pulse, and the fluence of a single laser pulse was 79 J/cm^2^. The laser beam was focused on the target using a cylindrical concave lens with a focal length of 10 cm. The incident angle of the laser beam was 90°. During the ablation, the targets were continuously scanned to avoid drilling. The detailed schematics and procedure of the two-step synthesis of doped nanoparticles can be found here [42]. Also, we measured the weight of ZnO substrates before and after the PLD and PLAL synthesis using a microbalance (XPR6UD5, Mettler Toledo, Columbus, OH, USA) to obtain the masses of deposited metal layers and ablated nanoparticles, respectively.

### 2.2. Material Characterization

The optical absorption properties of the X: ZnO (X-Ag, Au, Pt) nanoparticles in the colloidal solution were obtained via a UV-Vis spectrophotometer (Lambda 25, Perkin Elmer, Waltham, MA, USA) in the wavelength range from 220–800 nm. The UV-Vis absorption spectra were measured for nanoparticle colloidal solutions using a UV cuvette with a path length of 10 mm. Furthermore, optical properties were observed by photoluminescence (PL) spectroscopy. The photoluminescence measurements were obtained using a spectrofluorophotometer (RF-6000, Shimadzu, Kyoto, Japan) under an Xe lamp at the excitation energy of 3.54 eV (wavelength of 350 nm). Moreover, the colloidal solutions were dropped on silicon wafers and dried in a dryer (SP-25 Easy, Kambič, Semič, Slovenia) at 40 °C for 30 min for further characterization.

Structural analysis was carried out with the grazing incidence X-ray diffraction (GIXRD) technique. The measurements were performed in a diffractometer containing a Cu X-ray (λ = 1.5406 Å) tube and a W/C multilayer for monochromatization and beam shaping (D5000, Siemens, Karlsruhe, Germany). A curved position sensitive detector (RADICON) collected the diffracted spectra in the angular range 2θ = 30–85°. For all measurements, we used a fixed grazing incidence angle of $\alpha_{i}$ = 1.5°. The following JCPDS cards were used for crystallographic analysis: JCPDS 36-1451 (ZnO), JCPDS 04-0783 (Ag), JCPDS 04-0784 (Au), and JCPDS 01-087-0640 (Pt).

The detailed morphology and size distribution of the nanoparticles were studied using a scanning electron microscope (SEM, Joel 7600F, Tokyo, Japan) and a transmission electron microscope (TEM, JEOL JEM-1400 Flash, Tokyo, Japan). The SEM measurements were obtained by dropping a colloidal solution on a 5 × 5 mm^2^ Si wafer, while for the TEM images, samples were prepared by dropping one drop of a colloidal solution onto the TEM grid. All colloidal solutions were sonicated for 5 min before the dropping.

The chemical compositions were analyzed using an X-ray photoelectron spectroscope (PHI-TFA XPS, Physical Electronics Inc., Chanhassen, MN, USA) equipped with a monochromatic Al source at the photon energy of 1486.6 eV. The analyzed area and depth were 0.4 mm (in diameter) and 3–5 nm, respectively. The high-energy resolution spectrum was obtained with an energy analyzer, operating at a resolution of about 0.6 eV and pass energy of 29 eV. During data analysis, the spectrum was calibrated by setting the C 1s peak at 284.8 eV, characteristic of the C-C/C-H bonds. Quantification of surface composition was performed from XPS peak intensities, considering the relative sensitivity factors provided by the instrument manufacturer [43].

## 3. Results and Discussion

### 3.1. Mass and Atomic Fractions

Weighing the doped ZnO target before and after laser ablation reveals the mass and atomic fractions of dopants in the ZnO matrix. Table 1 shows masses of deposited Ag, Au, and Pt thin film on ZnO substrates. Pulsed laser deposition was the most effective for Ag thin film. The thickest film deposits in the case of silver. The amount of ablated material depends on the composition, geometry, and ablation threshold of the target, the focus and wavelength of the laser, the number of pulses, and the surrounding fluid in which the ablation takes place [34]. The pulsed laser ablation process was the most effective for Pt-doped ZnO nanoparticles, producing 1.925 mg in 6 min and 40 s. However, in this paper, we are focused on 2000 pulses (6 min and 40 s) for all three samples. From the obtained masses, we calculated the mass and atomic fractions of Ag, Au, and Pt in relation to the ZnO. The atomic fractions of Ag, Au, and Pt in ZnO were 2.32, 0.55, and 0.41%, respectively. Since the band-gap tuning of a semiconductor depends on the atomic fraction of impurity introduced into the crystal lattice, we can conclude that in this case, Ag would have the most effect on the band-gap narrowing.

### 3.2. Optical Analysis

**Figure 2:**
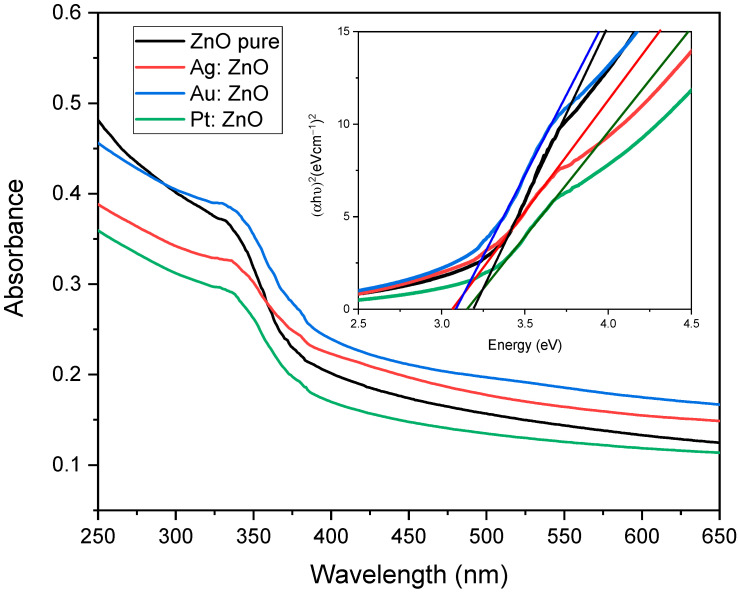
UV-Vis absorption spectra of the pure and Ag, Au, and Pt doped ZnO nanoparticles. The inset represents the Tauc plot of the same spectra showing the band-gap energies of 3.06 eV, 3.08 eV, 3.15 eV, and 3.20 eV for the Ag: ZnO, Au: ZnO, Pt: ZnO, and pure ZnO, respectively.

Using the UV-Vis spectrophotometer, we obtained the absorption spectra of the samples and extracted information about the optical band-gap energy. Figure 2 shows the UV-Vis absorption spectra of the pure and Ag, Au, and Pt doped ZnO nanoparticles in colloidal solution. We can distinguish characteristic ZnO peaks in the UV region between 300–400 nm due to intrinsic absorption when electrons transition from the valence band to the conduction band (O_2p_→Zn_3d_) [44]. By introducing Ag, Au, and Pt into the ZnO lattice, we have a broad absorption peak at about 335, 331, and 328 nm, respectively. We observed a red-shift of the Ag: ZnO and Au: ZnO NP absorption peaks compared to the Pt: ZnO peak. This implies a narrower optical band-gap in the case of Ag- and Au-doped ZnO [45]. A red-shift of the absorption peak can be connected to the development of shallow levels inside the band-gap due to the presence of impurity atoms present in the ZnO lattice [46]. From Table 1, we can notice that the highest atomic content of dopant in ZnO is in the case of Ag. This strongly indicates that Ag-doped ZnO will have the narrowest band-gap. Moreover, we do not see characteristic peaks of the Ag, Au, and Pt in ZnO spectrums, signifying the fact that these metals are incorporated into the crystal lattice of ZnO. We have different intensities in absorbance due to different solution densities.

Direct band-gap energies were obtained by extrapolating the linear part in a plot (α*hν*)^2^ versus *E_g_* = *hν* (inset in Figure 2) following equation:

(1) $${\left(\alpha h\nu \right)}^{2}=A\left(h\nu -E_{g}\right)\;$$

where *α* is the absorption coefficient, *h* is Planck’s constant, *hν* is the photon energy, *A* is a constant, and *E_g_* is the optical band-gap. The estimated band-gap energies are 3.06 ± 0.02 eV, 3.08 ± 0.02 eV, 3.15 ± 0.03 eV, and 3.20 ± 0.04 eV for the Ag: ZnO, Au: ZnO, Pt: ZnO, and pure ZnO, respectively. There is a decrease in the band-gap energies in the case of the doped ZnO NPs showing changes in the nanocrystal electronic structure of the doped ZnO [46] and more efficient light absorption. The narrowest band-gap energy has Ag-doped ZnO NPs because of the highest atomic content inside ZnO. Also, our synthesized ZnO has lower band-gap energy compared to the literature value of 3.37 eV [27].

**Figure 3:**
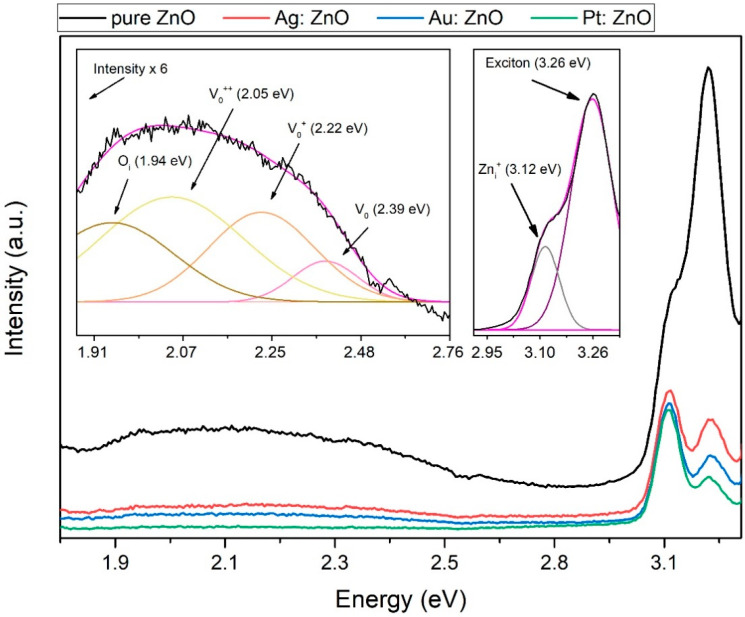
The photoluminescence (PL) spectra of pure and Ag, Au, and Pt doped ZnO NPs. In inset: Deconvolution plot in UV and visible part of PL spectrum (magenta line represent cumulative fit peak).

The photoluminescence (PL) spectra (Figure 3) obtained at room temperature offer an insight into the optical properties of the pure and doped ZnO NPs. Typical PL ZnO spectra consist of the peaks in the UV region related to the near-band-edge emission and the visible region attributed to the deep-level emissions. The peak at 3.26 eV (380 nm) for pure and doped ZnO is ascribed to the recombination of the electron and hole pairs (exciton recombination) aligning with the near-band edge of ZnO [47,48]. For doped ZnO NPs, the peak decreases compared to the pure ZnO, implying a decrease in the recombination rate of the electrons and holes. The emission peak at 3.12 eV (398 nm) can be attributed to the electronic transition from a lower energy excitonic state or to the Zn interstitial, which lies ~0.22 eV below the conduction band [49]. As it can be seen from the inset in Figure 3, the emission peak at 3.12 eV is well fitted with Zn_i_ interstitial [50]. Pure ZnO displays a yellow emission with a maximum at ~2.11 eV (~588 nm), which is related to the exciton transition between charged oxygen vacancy in the valence band and the photo-accelerated electrons [51]. To understand the origin of such a broad emission, the fit with several Gaussian components was performed. It was found that all peaks are related to the emission from ZnO, while the doped ZnO emission was too low to be fitted. In the inset in Figure 3, a deconvolution of the visible peak (yellow emission) for pure ZnO is shown [50]. It is deconvoluted into four Gaussian sub-peaks assigned to oxygen interstitial O_i_, oxygen vacancy double charge V_O_^++^, oxygen vacancy single charge V_O_^+,^ and oxygen vacancy V_O_ with the following shares in the total emission 26%, 42%, 24%, and 8%, respectively. The calculation of shares for V_O_^++^ and O_i_ is done by extrapolating Gaussian fits, as they are obstructed by the second order of initial irradiation, which appears below 1.9 eV (the peak is at 1.77 eV). It was widely accepted that V_O_^++^ is mainly responsible for the yellow emission, as is the case here where it dominates over the emission of other defects [52]. Doping ZnO with Ag, Au, and Pt decreases the visible emission, implying a low defect concentration. As said, the decrease in the PL peaks indicates a lower recombination rate of the excitons and improves their optical properties. This happens because dopants create band levels that act as traps for charge carriers, thus decreasing the recombination rate of the electrons and holes. Comparing PL intensities, Pt-doped ZnO NPs have the lowest recombination rate of the excitons. Since the exciton recombination rate decreases with the ZnO doping, a better photocatalytic power of ZnO in the degradation of organic pollutants is expected [53].

### 3.3. Structural Analysis

**Figure 4:**
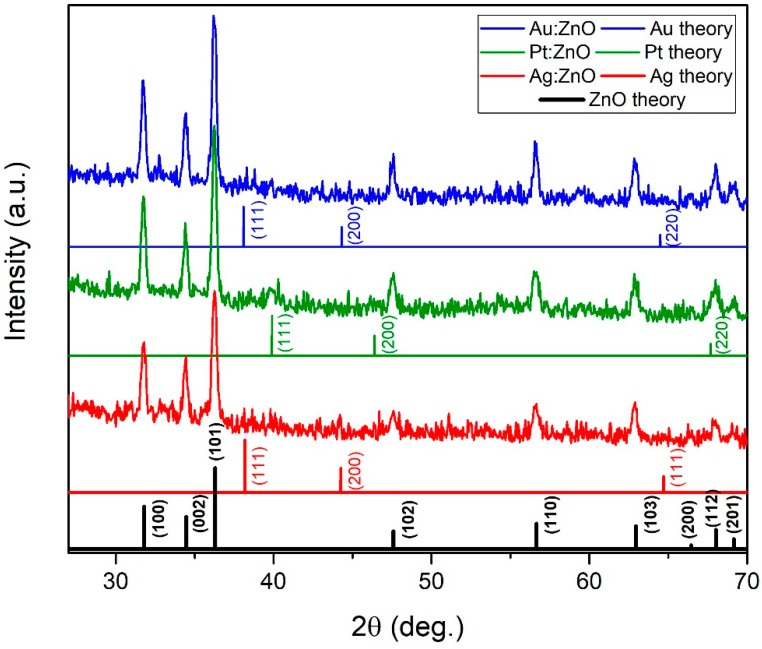
X-ray diffraction patterns of the Ag, Au, and Pt doped ZnO nanoparticles.

The XRD patterns reveal the crystal structure, purity, and crystallinity of the synthesized X: ZnO (X-Ag, Au, and Pt) nanoparticles. In Figure 4, all peaks are indexed as the hexagonal wurtzite structure of ZnO. The XRD peaks are relatively sharp indicating that X: ZnO nanoparticles are crystalline. Also, the XRD patterns are relatively broad because the crystals are randomly oriented, and we cannot exclude that a certain amount of amorphous ZnO and/or Zn(OH)_2_ is present in the main phase [44]. There are no characteristic peaks of impurities in the pattern. The absence of Au, Ag, and Pt characteristic peaks in the X: ZnO nanoparticles excludes the existence of the Au-, Ag-, and Pt-based clusters within the detection limit. This strongly implies that Ag, Au, and Pt have been incorporated into the crystal structure of ZnO and that high-quality doped nanoparticles were produced.

The average crystallite size (*D*) was calculated using Debye–Scherrer’s formula using data from the XRD patterns [54]:

(2) $$D=\frac{0.9\cdot \lambda }{\beta \cdot \cos\theta }$$

where *λ* is the wavelength of the X-ray beam (1.5406 Å), *β* is the full width at half maximum (in radians), and *θ* is the angle between the plane and the incident ray (Bragg’s angle, in radians). The calculated crystallite sizes are 50 nm, 24 nm, 25 nm, and 24 nm for pure ZnO, Ag: ZnO, Au: ZnO, and Pt: ZnO, respectively. Doped ZnO nanoparticles have smaller crystallites (half as small) compared to pure ZnO.

**Figure 5:**
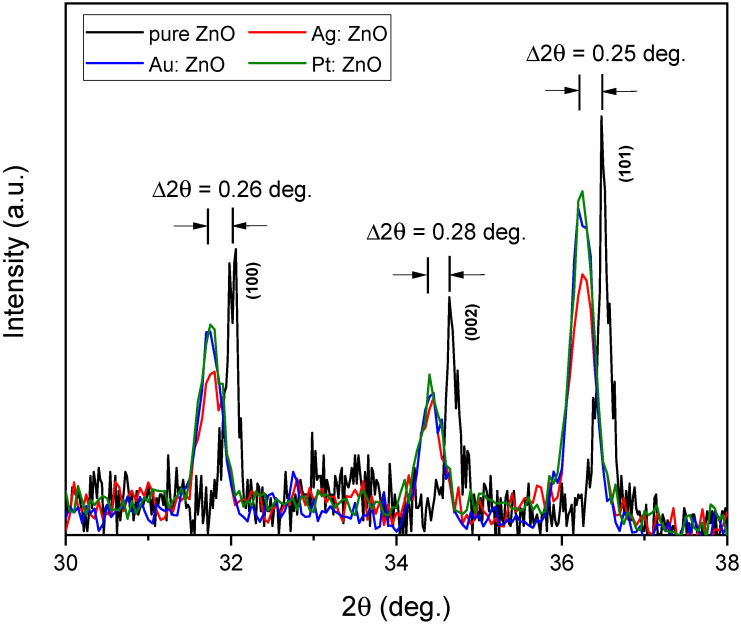
The Ag, Au, and Pt doped ZnO peak shifts compared to the pure ZnO peaks.

We observed shifts in the peaks of the hexagonal wurtzite structure (Figure 5) into smaller angles for the Ag: ZnO, Au: ZnO, and Pt: ZnO compared to the pure ZnO. These average shifts were 0.26° for Ag: ZnO, Au: ZnO, and Pt: ZnO. These shifts toward the smaller angles are attributed to the larger ionic radii of Ag^+^ (1.15 Å), Au^+^ (1.37 Å), and Pt^2+^ (0.8 Å) than Zn^2+^ (0.74 Å), which implies that Ag^+^, Au^+^, and Pt^2+^ substituted fraction of Zn^2+^ ions in the ZnO lattice. The sum of ionic radii (Ag + O, Au + O, Pt + O) is greater than the sum of ionic radii of Zn and O. Also, the bond lengths of Ag-O, Au-O, and Pt-O are longer than Zn-O in a hexagonal structure. This means that the unit cell should expand, and consequently, XRD peaks shift towards smaller angles. Similarly, Anugrahwidya et al. reported a shift in the main peak towards the smaller Bragg angles when Ag atoms substituted Zn atoms [39]. According to the literature, when a dopant has a bigger ionic radius than the matrix atom, then peaks shift towards smaller angles (e.g., Fe, Mn, In) [38,55,56]. On the contrary, when a dopant has a smaller ionic radius than the matrix atom, then peaks move towards higher values (e.g., Mg, Cu, Co, Ni) [35,37,55].

Lattice constants *a* (for (100) plane) and *c* (for (200) plane) can be calculated using the following formulae [54]:

(3) $$a=\frac{\lambda }{\sqrt{3}\cdot sin\theta }$$

(4) $$c=\frac{\lambda }{sin\theta }$$

From Table 2, it is apparent that the lattice constants *a* and *c* are larger for Ag-, Au-, and Pt-doped ZnO NPs compared to pure ZnO. With this calculation, we are confirming the lattice expansion of the doped ZnO NPs.

**Figure 6:**
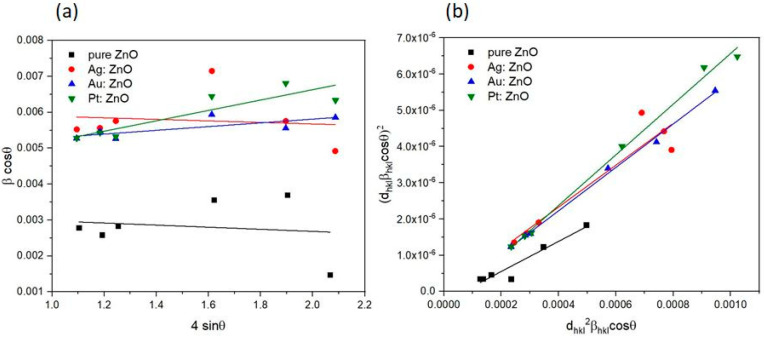
(**a**) Williamson-Hall and (**b**) size-strain analysis of pure and doped ZnO NPs. Using linear fit to the data, the crystallite size and strain are extracted and presented in Table 3.

The strain and average crystallite size can be determined from the Williamson-Hall (W-H) and strain-size (S-S) plots (Figure 6). The W-H plot (Figure 6a) uses the following relation:

(5) $$\left(\beta cos\theta \right)=\frac{0.94\lambda }{D}+\varepsilon \left(4sin\theta \right)$$

where *ε* is a microstrain. Crystallite size and microstrain are obtained from the intersection and slope value, respectively. The S-S plot (Figure 6b) is based on the relation:

(6) $${\left(d_{hkl}\beta_{hkl}cos\theta \right)}^{2}=\frac{0.94\lambda }{D}\left(d_{hkl}^{2}\beta_{hkl}cos\theta \right)+\frac{\varepsilon^{2}}{4}$$

where *d_hkl_* is interplanar spacing. Crystallite size and strain are obtained from the slope and intersection, respectively.

Table 3 presents the values of the crystallite size, strain, and dislocation density. Dislocation density is obtained using the following formulae [52]:

(7) $$\delta =\frac{1}{D^{2}}$$

From Table 3, it is apparent that the Ag:ZnO and Au:ZnO crystallite sizes obtained from three different methods are approximately the same, while for pure ZnO, a value between 35 and 50 nm is obtained. With Pt:ZnO, a larger crystallite size is obtained using the W-H method, which can give a larger number of crystallites because the broadening of the peak due to microstrains and crystallites is taken into account. Doped nanoparticles have a higher dislocation density than pure ZnO, which means that lattice defects occur and atoms in crystal cells are displaced from an ideal position due to the smaller crystallite size. The strains obtained for pure ZnO and Pt:ZnO are approximately the same, while for Ag:ZnO and Au:ZnO, there are deviations between the values.

### 3.4. Chemical Composition Analysis

To obtain further insight into the chemical composition and formation of doped ZnO nanoparticles, XPS analysis was applied to analyze the surface composition of such nanoparticles.

**Figure 7:**
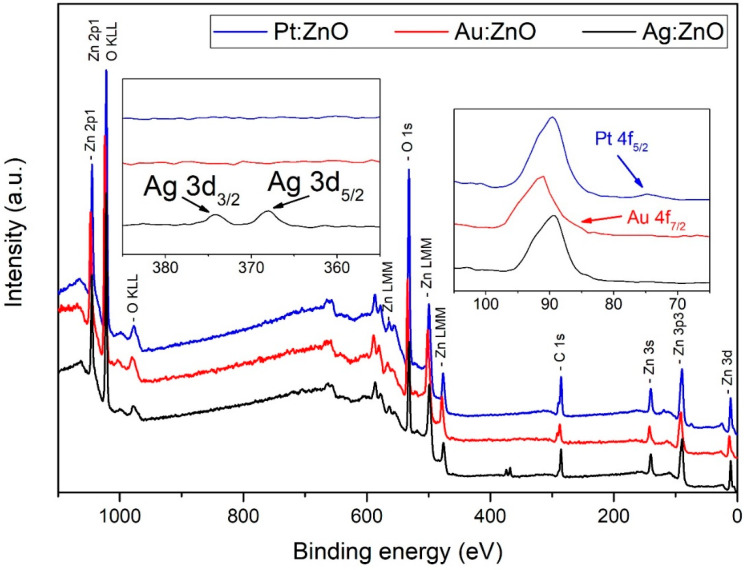
Wide XPS spectra of ZnO nanoparticles doped with Ag (black), Au (red), and Pt (blue). In insets: identification of Ag, Au, and Pt.

In Figure 7, wide XPS spectra are shown for ZnO nanoparticles doped with Ag, Au, and Pt. The presence of characteristic peaks can be identified for zinc (Zn 2p, Zn 3s, Zn 3p, Zn 3d, and Auger peaks Zn LMM), oxygen (O 1s and Auger peak O KLL), and carbon (C 1s). Peaks for Ag 3d, Au 4f, and Pt 4f can also be identified. Wide XPS spectra indicate that ZnO nanoparticles are doped and hence successfully synthesized.

**Figure 8:**
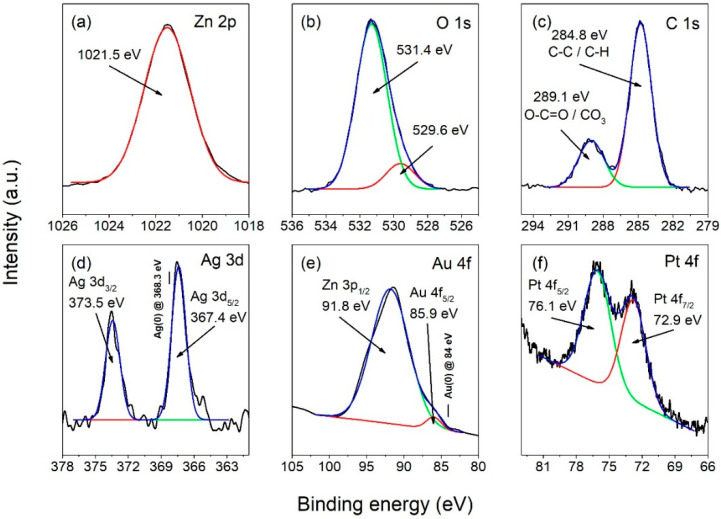
High resolution XPS spectra of doped ZnO nanoparticles with fitted spectra for (**a**) Zn 2p, (**b**) O 1s, (**c**) C 1s, (**d**) Ag 3d, (**e**) Au 4f, and (**f**) Pt 4f. Red and green curves represent fit peaks while blue curve represents cumulative fit peak.

The XPS high-resolution spectra are shown in Figure 8. Deconvolution of the Zn 2p_3/2_ peak (Figure 8a) reveals a gaussian distribution with a maximum at 1021.5 eV which corresponds to Zn^2+^ states in the ZnO crystal lattice [57]. Deconvolution of the O 1s (Figure 8b) peak results in two gaussian fits, first with a maximum at 529.6 eV and the second with a maximum at 531.4 eV. The first peak is related to a O^2−^ state that is built up in the ZnO crystal lattice, and the second peak is related to hydroxyl radical (O-H) [58]. Deconvolution of C 1s states (Figure 8c) results in two gaussian fits with maxima at 289.1 eV and 284.8 eV, related to C=O bonds and to C-C bonds, respectively [59]. Carbon occurs as a sample impurity, as samples were exposed to the atmosphere (C-H) after drying and before any analysis. The deconvolution of Ag 3d states (Figure 8d) resulted in two peaks at 373.5 eV and 367.4 eV related to 3d_3/2_ and 3d_5/2_ states, respectively. Those peaks are related to Ag-O bonding, as the same binding energies are characteristic for Ag_2_O [60]. The absence of a pure metallic Ag state expected at 368.3 eV (marked in spectrum) implies that all silver is incorporated in the ZnO lattice rather than in a separate nanoparticle form. The deconvolution of the Au 4f state (Figure 8e) resulted in two overlapping peaks at 91.8 eV (related to the Zn 3p_1/2_ state) and at 85.8 eV (related to the Au 4f_5/2_ state). The absence of the Au 4f_7/2_ state at 84 eV (marked in spectrum) implies that there is no Au in the metallic state and hence that Au is incorporated into the ZnO lattice rather than being attached to the ZnO NP surface as a pure nanoparticle [61,62,63]. The deconvolution of Pt 4f states (Figure 8f) resulted in two peaks at 76.1 eV and 72.9 eV, related to 4f_5/2_ and 4f_7/2_ states, respectively. Those states are related to metallic Pt, but they also exhibit a shift of 1.9 eV towards higher binding energies than that of pure metallic Pt [64]. This may be an indication that a Pt-Zn alloy is formed [65] beside the Pt incorporation into the ZnO lattice, as was revealed with XRD. It is known that Zn and Pt can be mixed together in a variety of different alloy compositions [66].

### 3.5. Morphological Analysis

**Figure 9:**
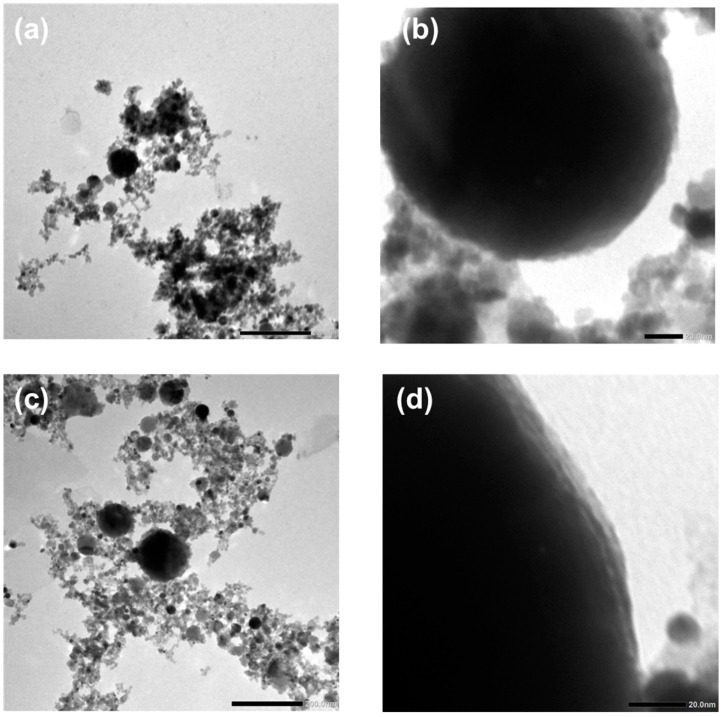
TEM images of (**a**,**b**) pure and (**c**,**d**) Pt-doped ZnO nanoparticles.

**Figure 10:**
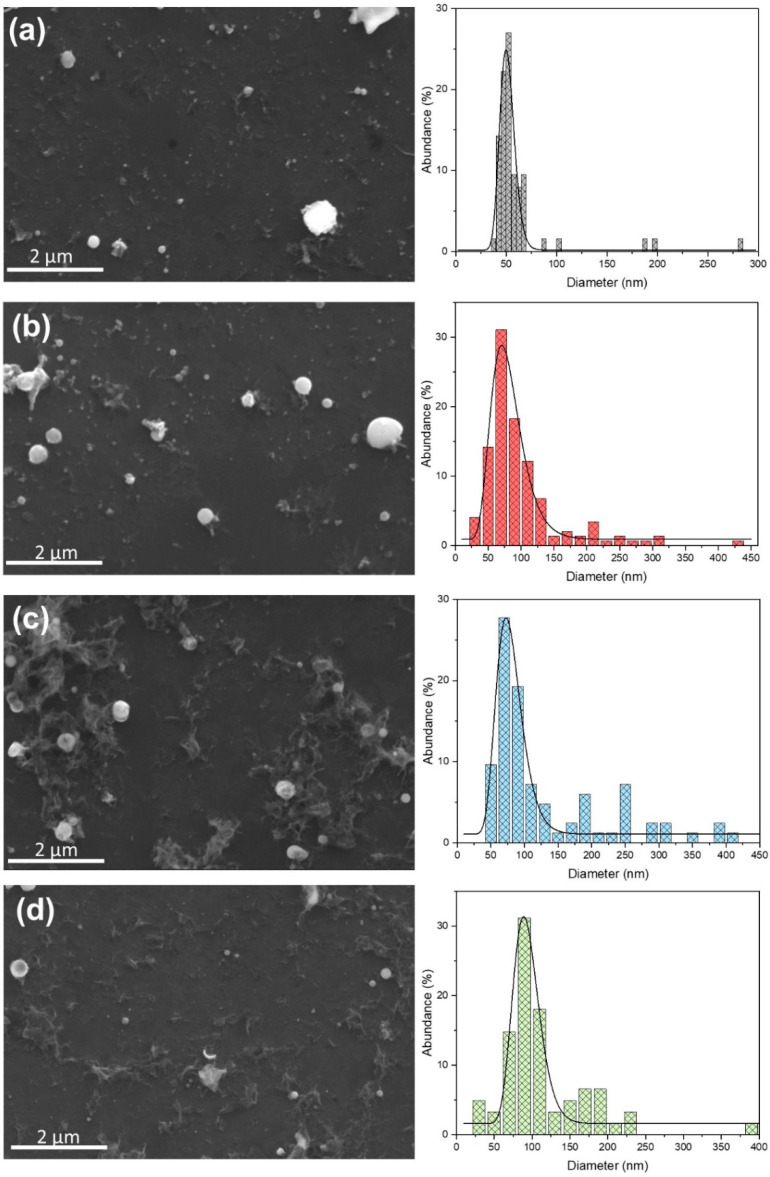
SEM images and size-distributions of (**a**) pure ZnO, (**b**) Ag-doped ZnO, (**c**) Au-doped ZnO, and (**d**) Pt-doped ZnO.

Morphological analysis (using TEM and SEM) of the samples revealed obtained nanoparticles and their size range. In Figure 9, TEM images for pure (a, b) and Pt-doped (c, d) ZnO NP are presented. The morphology of the nanoparticles is independent of the dopant material; therefore, we revealed TEM images of ZnO doped with gold (Figure 9c,d), which also describe the morphology of ZnO NPs doped with Ag and Pt. Figure 9a,c shows different-sized spherical nanoparticles and irregular material, which are formed during the laser ablation process. The surface morphology of the obtained NPs is shown in Figure 9b,d. The surface is relatively smooth with some roughness because the edges are not perfectly sharp. Figure 10 shows the SEM images of Ag-, Au-, and Pt-doped ZnO structures with their respective size distributions. Spherical nanoparticles from 50 nm up to 200 nm dominate in each sample. During the ablation process, some micro-sized particles and debris formed. Also, amorphous parts occurred, which agrees with the relatively broad XRD patterns. From SEM images, we determined the size distribution of nanoparticles. Size distribution is fitted as a log-normal function with maxima at diameters 51 nm, 71 nm, 73 nm, and 89 nm for the pure ZnO, Ag: ZnO, Au: ZnO, and Pt: ZnO, respectively. Pt-doped ZnO nanoparticles are slightly larger compared to the Ag- and Au-doped ZnO nanoparticles, while all doped ZnO NPs are larger than pure ZnO.

## 4. Conclusions

We demonstrated a novel two-step process using PLD and PLAL techniques for the production of Ag, Au, and Pt-doped ZnO NPs. The band-gap energies, calculated from the UV-Vis spectra, are 3.2 eV, 3.15 eV, 3.08 eV, and 3.06 eV for pure and silver, gold, and platinum-doped ZnO, respectively. The decrease in the band-gap energy implies changes in the nanocrystal electronic structure and more efficient light absorption. PL measurements showed that doped ZnO NPs have a lower recombination rate of the excitons compared to pure ZnO. Doped ZnO has no emission in the visible region compared to pure ZnO, implying low defect concentration. The XRD patterns showed that Ag-, Au-, and Pt-doped ZnO NPs maintained a hexagonal wurtzite structure without any Ag, Au, and Pt peaks. This, together with the fact that peaks are shifting towards smaller Bragg’s angles, confirms that Ag, Au, and Pt are well incorporated inside the ZnO lattice. It is also confirmed with the XPS study (to some minor extent only Pt appears in metallic form). Synthesized doped ZnO NPs are spherical-shaped with smooth surfaces, while morphology is independent of the dopant material. This study could provide a new way for the quick and clean synthesis of various doped materials.

## 5. Patents

Krstulović, N.; Blažeka, D.; Car, J.; Maletić, D.; Rakić, M. Method of Production of Two-Component Nanoparticles Using Laser. Croatian Patent P20211098A, 9 July 2021.

## Figures and Tables

**Table 1:** Mass measurements of deposited metal layers involved in ablation and ablated doped ZnO nanoparticles (NP). Also, the mass (w) and atomic (at.) fractions of Ag, Au, and Pt in the produced nanoparticles were calculated.

PLD		PLAL			w (%)	at. (%)
m (Ag)	0.036 mg	m (Ag: ZnO)	1.171 mg	Ag	3.37 ± 0.2	2.32 ± 0.2
m (Au)	0.024 mg	m (Au: ZnO)	1.798 mg	Au	1.33 ± 0.1	0.55 ± 0.1
m (Pt)	0.019 mg	m (Pt: ZnO)	1.925 mg	Pt	1.01 ± 0.1	0.42 ± 0.1

**Table 2:** Comparison of the calculated lattice constants a and c with the respected c/a ratio.

	a (Å)	c (Å)	c/a (Å)
pure ZnO	3.226	5.170	1.603
Ag: ZnO	3.250	5.207	1.602
Au: ZnO	3.252	5.206	1.601
Pt: ZnO	3.251	5.207	1.603

**Table 3:** Comparison of the obtained crystallite sizes (D) using the Debye–Scherer formula (D-S), Williamson-Hall (W-H), and size-strain (S-S) plots. Corresponding dislocation densities (δ) and strains (ε) are presented.

	D_D-S_ (nm)	D_W-H_ (nm)	D_S-S_ (nm)	δ_(D-S)__× 10_^−3^ (nm^−2^)	δ_(W-H)__ × 10_^−4^ (nm^−2^)	δ_(S-S)__ × 10_^−4^ (nm^−2^)	ε_(W-H) × 10_^−4^	ε_(S-S) × 10_^−4^
ZnO	50	44 ± 8	35 ± 6	0.04	0.05	0.08	1.4 ± 0.4	1.1 ± 0.7
Ag: ZnO	24	24 ± 5	25 ± 4	0.17	0.17	0.16	2.1 ± 0.1	0.2 ± 0.1
Au: ZnO	25	30 ± 7	24 ± 5	0.16	0.11	0.17	5.2 ± 2.3	0.8 ± 0.2
Pt: ZnO	24	39 ± 7	21 ± 6	0.17	0.06	0.23	1.5 ± 0.3	1.3 ± 0.4

## Data Availability

Data available on request.
